# Nonlinear association between wet-bulb globe temperature and maternal hypertensive disorders burden: a global analysis from 1990 to 2021

**DOI:** 10.3389/fpubh.2025.1678469

**Published:** 2025-10-10

**Authors:** Boya Zhao, Yonghui Zhao, Zhan Hu, Xin Duan, Jiujie Dou, Senlin Shi

**Affiliations:** ^1^Center for Reproductive Medicine, The First Affiliated Hospital of Zhengzhou University, Zhengzhou, China; ^2^Department of Orthopedics, The First Affiliated Hospital of Xi'an Jiaotong University, Xi'an, China

**Keywords:** global burden database, maternal hypertensive disease, wet-bulb globe temperature, restricted cubic spline, sociodemographic index

## Abstract

**Background:**

Maternal hypertensive disorders (MHD) are major causes of maternal mortality and perinatal complications worldwide, and their disability burden has risen with pronounced geographic disparities. Under global warming, heat stress is increasingly implicated in dysregulated blood pressure during pregnancy, yet robust global-scale evidence remains limited.

**Methods:**

We combined age-standardized years lived with disability (AS-YLDs) from MHD for 204 countries (1990–2021) with wet-bulb globe temperature (WBGT). Restricted cubic splines were used to characterize nonlinear WBGT–AS-YLDs associations. Analyses were stratified by five Sociodemographic Index (SDI) levels and adjusted for environmental and behavioral covariates (body-mass index, air pollution, tobacco use, alcohol consumption, iron deficiency) plus calendar-year fixed effects. Sensitivity analyses further stratified by climate zones.

**Results:**

Globally, WBGT showed a significant nonlinear association with MHD AS-YLDs, approximating a *U*-shaped curve. The lowest burden occurred near 11.7 °C, followed by increases beyond this point, a brief dip around 23.5 °C, and renewed rise above 27 °C. Similar *J*-/*U*-shaped patterns appeared in low–median, median, and high–median SDI strata, with modest shifts in inflection temperatures; low-SDI countries showed a pronounced *J*-shape, whereas high-SDI countries exhibited no clear nonlinearity. WBGT and SDI displayed stronger associations with MHD burden than other covariates. Year fixed effects were generally small and often non-significant. Results were consistent across climate-zone strata.

**Conclusion:**

This study quantifies a global, nonlinear relationship between WBGT and the MHD disability burden, with heterogeneous effects across development levels. The findings highlight heat exposure as a potential threat to maternal health under climate change and support targeted mitigation and adaptation—especially in low- and median-SDI settings.

## 1 Introduction

Maternal hypertensive disorders (MHD), including gestational hypertension, preeclampsia, eclampsia, chronic hypertension complicating pregnancy, and chronic hypertension with superimposed preeclampsia, represent one of the most prevalent and serious pregnancy complications. In severe cases, MHD can lead to intrauterine growth restriction, preterm birth, or even fetal death (1). The global prevalence of MHD is estimated at approximately 10%−22%, showing a continuous upward trend in recent years (2). Substantial evidence indicates that women with a history of hypertensive disorders during pregnancy are at increased risk of developing chronic hypertension, cardiovascular diseases, renal impairment, and even cognitive decline postpartum. Their offspring also face elevated risks of metabolic disorders, high blood pressure, and neurodevelopmental impairments (3, 4). In the United States, the incidence of MHD has exceeded 10% and continues to rise (5). Notably, the disease burden displays significant regional variations. These disparities are closely associated with socioeconomic development, healthcare accessibility, and maternal risk factors including advanced age, obesity, and multiple pregnancies.

Environmental factors are receiving increasing attention as potential contributors to MHD. In the context of global climate change, temperature fluctuations have been identified as a possible risk factor affecting maternal blood pressure regulation during pregnancy (6). Multiple systematic reviews and meta-analyses consistently indicate that maternal heat exposure is associated with an increased risk of hypertensive disorders of pregnancy (including preeclampsia and gestational hypertension), with stronger associations observed during the early to mid-pregnancy period (7). Regional cohort studies have indicated that exposure to extreme heat or cold during early pregnancy can increase the risk of gestational hypertension and preeclampsia (8, 9). Meta-analyses have also shown that heat exposure not only has immediate effects on maternal health and increases pregnancy-related risks, but is also linked to fetal growth and development as well as long-term outcomes in adulthood, including educational attainment, income level, and cardiovascular and respiratory health (10). In addition, both air quality and humidity have been associated with MHD outcomes (11, 12). However, there remains a lack of large-scale, systematic studies based on global datasets, particularly those utilizing comprehensive heat stress metrics such as Wet-Bulb Globe Temperature (WBGT) to assess their impact on disease burden indicators such as Years Lived with Disability (YLDs). Compared with using ambient temperature alone, reviews of heat indices have highlighted that comprehensive measures such as WBGT and UTCI better reflect the physiological burden of heat stress. Moreover, some studies have demonstrated that WBGT provides stable predictive performance in assessing disruptions in maternal healthcare utilization and adverse pregnancy outcomes (13).

In this study, we leveraged high-resolution climate data from the Google Earth Engine (GEE) platform and MHD burden data from the Global Burden of Disease (GBD) study (14), combined with age-standardized YLD rates and WBGT exposure data covering 200 countries and territories from 1990 to 2021. WBGT, which incorporates ambient temperature, humidity, wind speed, and solar radiation, more accurately reflects physiological heat load than temperature alone and has been widely applied in global monitoring of heat-related health risks (15).

Given the considerable differences in climate conditions, socioeconomic status, and healthcare infrastructure across countries, we hypothesize that the impact of WBGT on MHD burden may exhibit substantial heterogeneity, potentially explaining part of the global spatial variation in MHD observed in GBD data. To address this, our study employs a systematic and multi-regional approach to evaluate the association between WBGT and the health burden of maternal hypertensive disorders. Our findings aim to uncover the potential impact of climate factors on maternal and perinatal health and to inform public health interventions and early warning strategies for pregnancy-related disorders under global climate change.

## 2 Methods

### 2.1 Data collection and global heatmap construction

Age-standardized years lived with disability due to maternal hypertensive disorders Age-Standardized Years Lived with Disability (MHD AS-YLDs) were obtained from the Global Burden of Disease (GBD) 2021 database, covering the annual burden in women aged 10–54 years across 200 countries from 1990 to 2021. Wet-Bulb Globe Temperature (WBGT) data were sourced from 2 monthly datasets on the Google Earth Engine (GEE): (1) Climate Hazards Center (CHC) covering 1990–2016 (16), and (2) ERA5 (ECMWF Reanalysis v5) reanalysis data covering 1990–2021 (17). Both WBGT datasets were aggregated to population-weighted national annual means. To harmonize sources while preserving temporal trends, we learned a country-specific quantile-delta mapping (QDM) between ERA5 and CHC over the overlap period (1990–2016) and applied it to ERA5 for 2017–2021 (18, 19); the corrected ERA5 series was then concatenated with CHC to produce a continuous 1990–2021 record. We visualized the results as global heatmaps using stratified color scales for MHD AS-YLDs and harmonized WBGT, with zoomed-in panels for key regions. We classified the 200 countries included in the GBD database into five Socio-demographic Index (SDI) categories—low, low-median, median, high-median, and high—according to the IHME (Institute for Health Metrics and Evaluation)-GBD methodology (20). MHD AS-YLDs data were obtained from the GBD database as annual age-standardized YLDs for maternal hypertensive disorders for each country. Among the exposure variables, we used age-standardized SEV (Summary Exposure Value) data for all-age females only for nitrogen dioxide (NO_2_); for all other exposures, SEV estimates were restricted to females aged 10–55 years and subsequently age-standardized. Table 1 presents the mean values and standard deviations of all outcome, exposure, and covariate variables averaged across country-years within each SDI group.

**Table 1:** Mean values (standard deviations) of key environmental and behavioral exposures and health burden of maternal hypertensive disorders across five sociodemographic index (SDI) groups and globally, 1990–2021.

**SDI group**	**WBGT (°C)**	**MHD (AS-YLDs)**	**High BMI (SEV)**	**Iron def. (SEV)**	**Tobacco (SEV)**	**Alcohol (SEV)**	**Ozone (SEV)**	**NO_2_ (SEV)**
Global	20.01 (8.69)	458.06 (488.75)	23.19 (10.52)	12.55 (1.51)	26.71 (11.03)	17.92 (14.75)	12.56 (9.73)	16.38 (18.79)
High	11.98 (8.91)	128.86 (75.73)	24.83 (8.47)	11.85 (2.12)	33.14 (7.59)	34.46 (13.06)	13.88 (9.33)	34.75 (22.88)
High-median	17.81 (9.33)	203.46 (105.33)	28.46 (10.02)	12.55 (1.19)	30.7 (12.11)	20.16 (13.14)	12.2 (9.8)	14.4 (14.16)
Low	25.82 (2.79)	1,211.37 (440.61)	13.68 (5.72)	13.26 (0.7)	18.06 (8.26)	7.66 (5.49)	9.92 (8.2)	3.32 (3.47)
Low-median	22.57 (6.85)	565.34 (419.93)	24.25 (13.2)	12.89 (1.1)	22.26 (9.96)	8.57 (6.23)	12.95 (10.41)	7.54 (7.54)
Median	21.61 (6.74)	257.91 (140.29)	25.09 (8.31)	12.49 (1.25)	27.31 (9.74)	12.35 (8.94)	13.46 (10.47)	14.68 (12.82)

Values within cells represent means and corresponding standard deviations (in parentheses) for each exposure and outcome, stratified by SDI group and global level.

### 2.2 Histogram of annual average WBGT distribution across countries

WBGT values from 1990 to 2021 were grouped into five equal-width intervals for all 200 countries. Within each WBGT category, boxplots were created to display the distribution of MHD AS-YLDs. These boxplots were constructed separately for each Sociodemographic Index (SDI) group (low, low–median, median, high–median, high) and for the global aggregate. The *x*-axis represented the WBGT categories (°C), and the *y*-axis indicated the MHD AS-YLDs within each corresponding interval.

### 2.3 RCS curves of WBGT and MHD burden across SDI groups, climate zone, and globally

To evaluate the shape of the association between WBGT and MHD burden, restricted cubic spline (RCS) functions were used to model WBGT, and results were compared with traditional linear models (21). In each RCS model, MHD AS-YLDs served as the dependent variable, with WBGT modeled both linearly and using splines, controlling for covariates such as BMI (Body-Mass Index), smoking, alcohol, and pollution exposure. In the SDI and climate zone-stratified models, the restricted cubic spline was specified with three knots at the 10th, 50th, and 90th percentiles (0.10, 0.50, 0.90) owing to the smaller sample size within each group. To ensure stability of the spline models, we restricted climate zone to those containing more than six countries. This threshold was chosen because strata with fewer countries showed insufficient between-country variation in WBGT, leading to unstable spline fits dominated by within-country year-to-year fluctuations.

By contrast, the global RCS model used four knots at the 5th, 35th, 65th, and 95th percentiles (0.05, 0.35, 0.65, 0.95). *F*-tests were used to compare residual sum of squares (RSS) between models to assess improvements in model fit. For the main RCS model, the dependent variable was log(MHD AS-YLDs + 1e−6), and WBGT was modeled using restricted cubic splines. Models were fitted separately for each SDI group and for the global dataset. With covariates held constant at reference levels, 200 evenly spaced values of WBGT within each group-specific distribution were used to generate fitted values and 95% confidence intervals. The first and second derivatives of the fitted curves were analyzed to identify local minima and maxima, which were then labeled on the plots. We used cleaned 1990–2021 data, excluded missing values. The outcome was log-transformed MHD AS-YLDs. All models adjusted for SDI, key metabolic and environmental risk factors, and year. Linear, quadratic, polynomial, RCS (3- and 4-knot), and GAM (Generalized Additive Model) models were compared using AIC/BIC to assess model fit.

### 2.4 Covariate association estimation based on RCS models

RCS models were further used to evaluate the association between various covariates and MHD burden (MHD AS-YLDs). Independent variables included spline terms for WBGT (four knots), fixed effects for year (with 1990 as reference), behavioral and environmental exposures (e.g., BMI, tobacco use, alcohol consumption, iron deficiency NO_2_ and ambient ozone pollution), and SDI. The results were presented as percent changes in MHD AS-YLDs per unit increase in each covariate, along with corresponding 95% confidence intervals.

### 2.5 Fixed effects modeling of calendar year based on RCS framework

To estimate the temporal fixed effects of calendar year on MHD AS-YLDs, generalized estimating equations (GEE) were applied, controlling for multiple environmental and behavioral covariates (22, 23). Analyses were performed separately for each SDI group (low, low–median, median, high–median, high) and globally. Using 1990 as the reference year, we plotted the annual percent change in MHD AS-YLDs relative to 1990. In the resulting figures, the blue line represents point estimates, and the golden shaded area indicates the 95% confidence intervals.

### 2.6 Collinearity diagnostics

To assess potential multicollinearity among covariates included in the regression model, we calculated variance inflation factors (VIFs) for all covariates. A multiple linear regression model was constructed with log(MHD-YLDs + 1e−6) as the dependent variable and the following variables as predictors: Socio-demographic Index (SDI), high body-mass index, iron deficiency, tobacco use, high alcohol consumption, ambient ozone pollution, particulate matter pollution, and nitrogen dioxide pollution. VIF values >10 were considered indicative of serious multicollinearity.

### 2.7 Heterogeneity by climate zones

To assess heterogeneity, within each SDI stratum we further stratified countries by climate zone (Cold/Polar, Temperate, Subtropical, Tropical) and fitted RCS–GEE models identical to the main analysis. WBGT was modeled with restricted cubic splines, with knots placed at within-stratum quantiles, adjusting for BMI, iron deficiency, tobacco use, alcohol consumption, particulate matter, nitrogen dioxide, and calendar-year fixed effects. We reported zone-specific exposure–response curves with 95% confidence intervals, and in pooled models included WBGT-spline × climate-zone interaction terms; joint Wald tests were used to evaluate differences across climate zones.

## 3 Results

### 3.1 Global distribution of MHD AS-YLDs and WBGT averages from 1990 to 2021

We first harmonized WBGT data from two sources—CHC (1990–2016) and ERA5 (2017–2021)—using quantile delta mapping (QDM) to adjust for systematic biases. The two series were then merged into a continuous country-level dataset spanning 1990–2021. To verify splicing consistency, we compared WBGT values between 2016 (CHC) and 2017 (bias-adjusted ERA5), and the distribution of inter-annual differences showed no abrupt jumps (Supplementary Figure 1). This confirms that our QDM-based bias correction effectively eliminated discontinuities across data sources.

**Figure 1:**
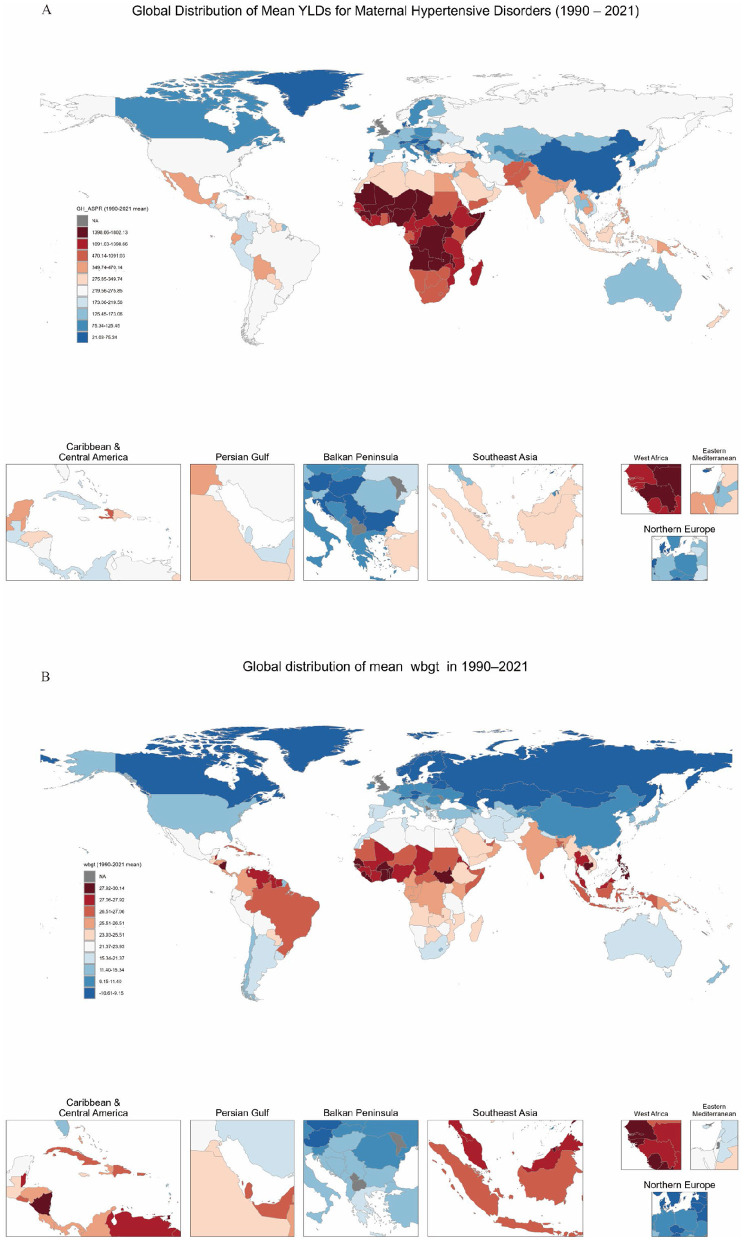
Global distribution of maternal hypertensive disorders (MHD) AS-YLD rates and WBGT, 1990–2021. **(A)** Shows the global distribution of age-standardized years lived with disability (AS-YLDs) due to maternal hypertensive disorders across 200 countries from 1990 to 2021. **(B)** Visualizes the corresponding national average wet-bulb globe temperature (WBGT) over the same period. In both maps, red indicates higher values (disease burden or temperature), while blue indicates lower values. Selected countries and regions are labeled on the maps for reference.

Subsequently, we constructed global heatmaps for the mean WBGT and MHD AS-YLDs. High MHD burden was concentrated in sub-Saharan Africa and parts of the Middle East, with average burdens exceeding 400 per 100,000 in many regions and surpassing 800 per 100,000 in several countries. In contrast, North America, Western Europe, China, and Australasia showed significantly lower burdens (Figure 1A). Globally, WBGT exhibited a strong latitudinal gradient: values exceeded 25 °C in equatorial regions, reaching over 27 °C in parts of the Sahara and South Asia, while high-latitude areas such as Northern Europe, Russia, and Canada recorded WBGT below 10 °C (Figure 1B). The spatial overlap between high WBGT and high MHD AS-YLDs in Africa and South Asia suggests a potential association between heat exposure and disease burden (20).

Grouped by SDI levels, we found the global mean WBGT was 20.01 °C (SD = 8.69), with the highest in low SDI countries (25.82 °C, SD = 2.79) and the lowest in high SDI countries (11.98 °C, SD = 8.91). MHD AS-YLDs were highest in low SDI countries (1,211.37) and lowest in high SDI countries (128.86). The prevalence of high BMI (SEV) peaked in high–median countries (28.46) and was notably lower in low SDI countries (13.68). Tobacco and alcohol use were markedly higher in high SDI countries, at 33.14 and 34.46, respectively. NO_2_ was highest in high SDI countries (34.75), indicating substantial disparities in environmental pollution across SDI groups (Table 1).

### 3.2 Distribution of MHD AS-YLDs across WBGT groups

**Figure 2:**
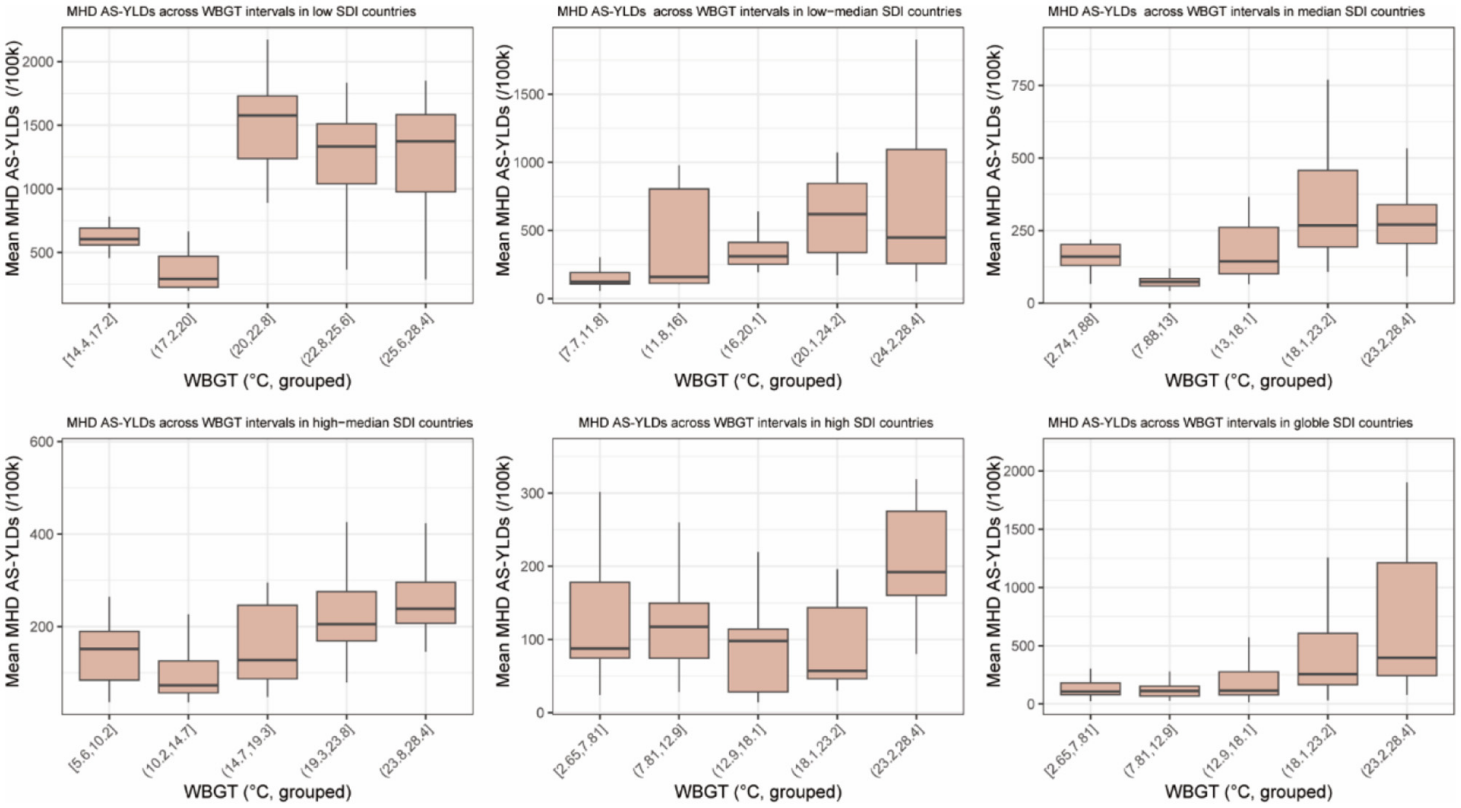
Boxplots of age-standardized YLDs for maternal hypertensive disorders (MHD AS-YLDs) across WBGT groups. The figure comprises six subplots corresponding to the five sociodemographic index (SDI) categories (low, low–median, median, high–median, and high) and the global level. The *x*-axis represents WBGT (°C), grouped into equal-width intervals. The *y*-axis shows MHD AS-YLDs (age-standardized years lived with disability for maternal hypertensive disorders, per 100,000 population) within each WBGT interval.

Boxplots stratified by SDI groups revealed differing trends in MHD AS-YLDs across WBGT intervals. In low SDI countries, MHD AS-YLDs were substantially higher in WBGT ranges ≥ 20 °C, with wide interquartile ranges and numerous outliers, suggesting high variability. Both low–median and median SDI groups also exhibited positive associations between WBGT and MHD burden, though with gentler slopes. In the high–median group, overall MHD AS-YLD levels were lower, but an upward trend was still evident. For high SDI countries, countries in 23.2 to 28.4 show a higher MHD AS-YLDs (Figure 2).

At the global level, median MHD AS-YLDs increased steadily across WBGT intervals, indicating a consistent exposure–burden relationship and supporting the hypothesis that heat exposure may be linked to maternal hypertensive burden.

### 3.3 *U*-shaped association between WBGT and MHD AS-YLDs

To further investigate the relationship between WBGT and maternal hypertensive disorders, we first compared model performance. Restricted cubic spline (RCS) models significantly reduced residual sum of squares (RSS) compared to linear models across all five SDI groups and the global level (Global: *F* = 114.1, *p* < 0.001), with the most substantial improvements observed in high–median (*F* = 94.0) and median SDI countries (*F* = 100.0; Supplementary Table 1). AIC validation indicated that regardless of whether knots were set to 3 or 4, RCS models outperformed GAM and polynomial models (Supplementary Table 2). These results indicate a complex, nonlinear association between WBGT and MHD burden, justifying the use of RCS models for further analysis (24).

The fitted RCS models revealed significant nonlinear relationships between WBGT and MHD AS-YLDs at both global and regional levels, displaying *U*-shaped or *J*-shaped patterns. Globally, the minimum disease burden occurred at ~11.1 °C WBGT. As WBGT increased to 24.0 °C, MHD AS-YLDs rose to a peak (335.01 per 100,000), followed by a decline to a local minimum at 27.2 °C (256.31 per 100,000), and then rebounded upward.

**Figure 3:**
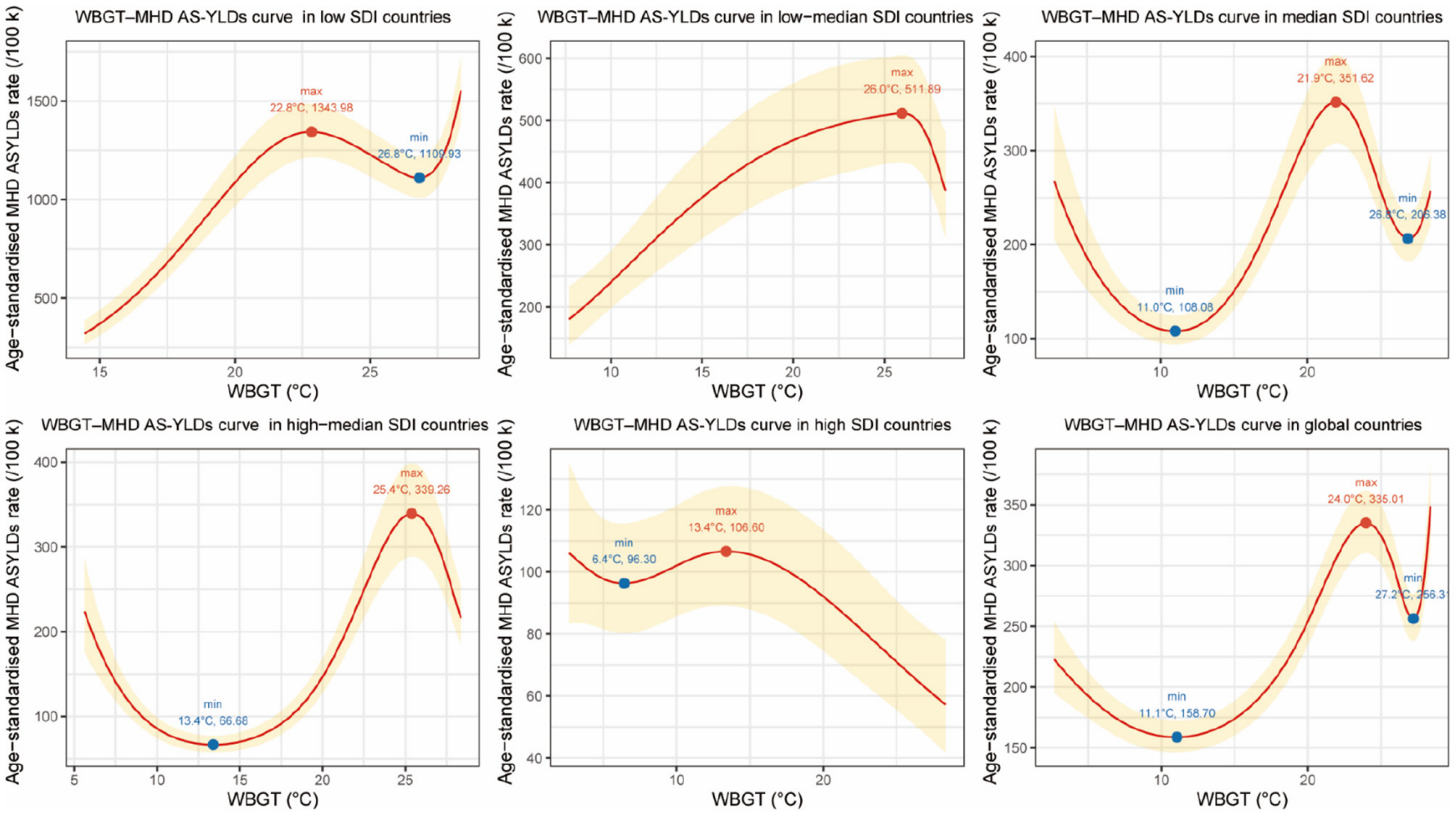
Restricted cubic spline (RCS) curves for the association between wet-bulb globe temperature (WBGT, °C) and age-standardized YLDs for maternal hypertensive disorders (MHD AS-YLDs, per 100,000) across SDI groups and globally, 1990–2021. The figure presents six panels representing the five sociodemographic index (SDI) groups (low, low–median, median, high–median, high) and the global level. The *x*-axis denotes annual mean WBGT (°C), and the *y*-axis indicates MHD AS-YLDs. Red solid lines represent multivariable-adjusted RCS fitted values, with golden shaded areas indicating 95% confidence intervals. Solid blue or red dots along the curves mark inflection points where the first derivative changes sign, corresponding to local minima or maxima in MHD burden. Text labels show the WBGT temperature at which the slope changes direction and the estimated MHD AS-YLDs at that point. These inflection points highlight critical WBGT thresholds where the health risk either starts to increase or decreases, helping to identify temperature ranges most strongly associated with maternal hypertensive disorder burden.

In low SDI countries (WBGT >14 °C), a *J*-shaped pattern emerged with a burden peak at 22.8 °C (1,343.98 per 100,000), a subsequent decrease, and a renewed rise beyond 26.8 °C. In low-median SDI countries, MHD AS-YLDs exhibited a positive association with WBGT, peaking at ~26.0 °C. In median and high–median SDI countries, burden minima were observed at 11.0 and 13.4 °C, respectively, followed by a steady rise, peaking at 21.9 and 25.4 °C. Notably, in median SDI countries, the burden continued increasing beyond 26.8 °C. In high SDI countries, the association between WBGT and MHD burden was less apparent, potentially due to effective heat-mitigation strategies. Narrow 95% confidence intervals across regions suggest robust model stability (Figure 3). These findings underscore the significant risk posed by extreme temperatures—especially elevated WBGT—on hypertensive disorders during pregnancy, with variations across development levels. Region-specific heat-related maternal health interventions are warranted.

To complement the RCS analysis, a forest plot was generated showing the effect estimates of the WBGT spline terms. Knots 1 to 5 were significantly positively associated with MHD burden. The overall RCS model clearly outperformed the linear alternative. SDI showed a strong inverse association with MHD AS-YLDs, indicating a protective effect of higher socioeconomic development. Other covariates, including BMI, iron deficiency, tobacco, and alcohol use, had limited or non-significant effects. O_3_ and NO_2_ pollution showed a marginally negative but unstable relationship (Supplementary Figure 2). Overall, the fully adjusted GEE models (Table 2) indicated that WBGT spline terms and SDI were the primary drivers of global variation in MHD AS-YLDs. WBGT showed significant positive associations across multiple spline knots (% change range from 42.04 to 141.28), whereas SDI displayed a strong inverse effect (% change −96.75%). In contrast, other behavioral and environmental covariates (BMI, iron deficiency, tobacco, alcohol use, O3, and NO_2_) exhibited smaller or non-significant associations.

**Table 2:** GEE-estimated percent change.

**Variable**	**% change**	**Lower 95% CI**	**Upper 95% CI**	***p*-Value**
ns (wbgt, knots = knots) 1	102.76	52.74	169.16	1.01E-06
ns (wbgt, knots = knots) 2	81.61	29.41	154.86	5.59E-04
ns (wbgt, knots = knots) 3	65.61	28.41	113.58	1.02E-04
ns (wbgt, knots = knots) 4	141.28	59.15	265.79	3.34E-05
ns (wbgt, knots = knots) 5	42.04	9.15	84.85	0.009026093
sdi	−92.84	−96.54	−85.18	1.22E-12
High body-mass index	−0.54	−1.41	0.34	0.229046666
Iron deficiency	−0.5	−5.1	4.32	0.834881435
Tobacco	−0.43	−1.05	0.19	0.174080389
High alcohol use	−0.19	−1.12	0.75	0.689257436
Ambient ozone pollution	−0.71	−1.11	−0.32	3.81E-04
Nitrogen dioxide pollution	−0.27	−0.47	−0.06	0.009845495

All covariates exhibited VIF values below 5, indicating no evidence of severe multicollinearity among the variables. This supports the inclusion of these covariates in the multivariable regression model for adjusted analyses (Supplementary Table 3).

### 3.4 Calendar year effects were not significant in most countries

**Figure 4:**
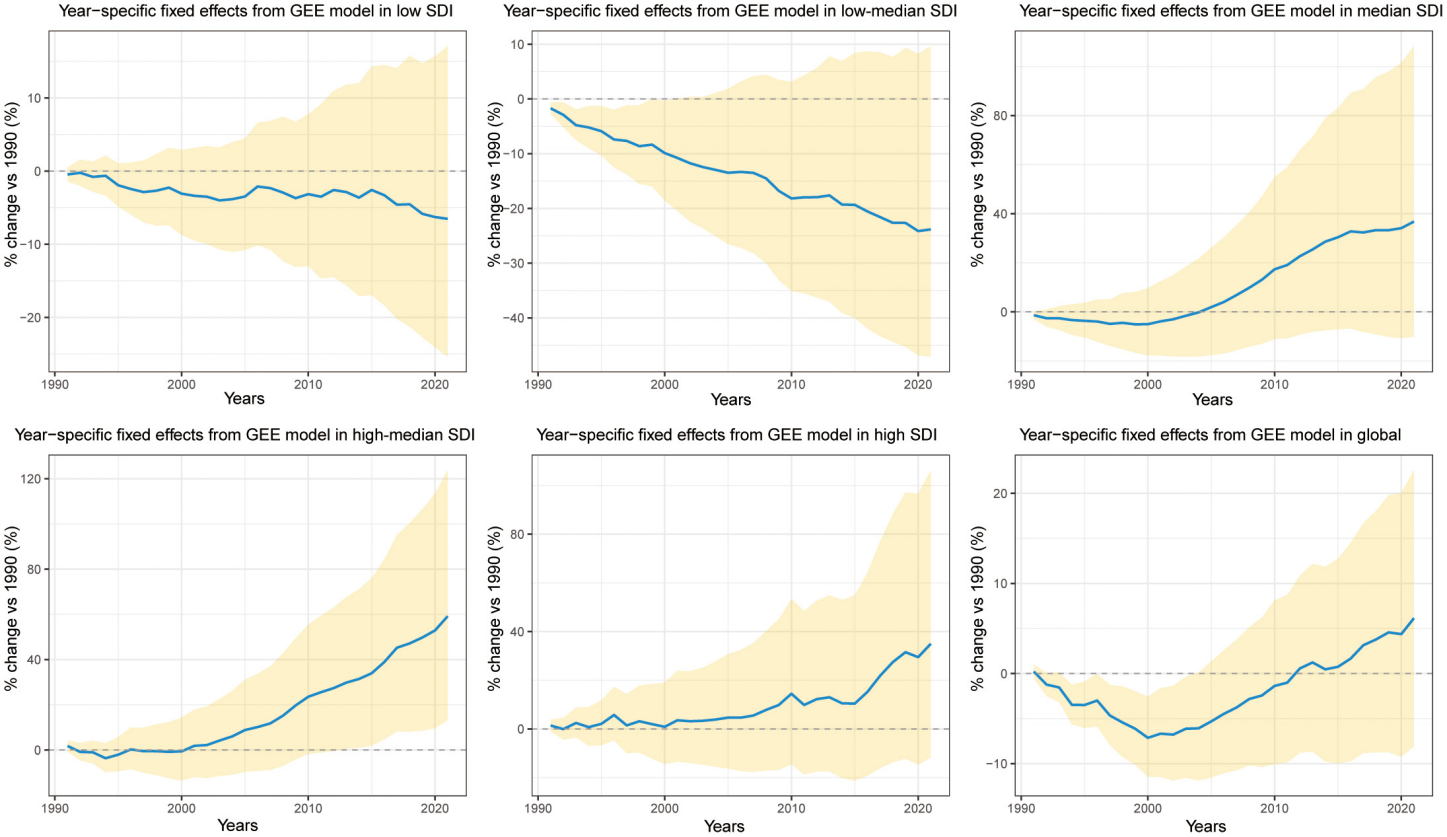
Trends in year fixed effects (% change relative to 1990) for age-standardized YLDs of maternal hypertensive disorders (MHD AS-YLDs) across SDI groups and globally, 1990–2021. The figure contains six subplots representing the five sociodemographic index (SDI) groups (low, low–median, median, high–median, high) and the global level. The *x*-axis indicates calendar years, and the *y*-axis shows the percent change in MHD AS-YLDs relative to the baseline year 1990. Solid blue lines represent point estimates of year fixed effects; golden shaded areas denote 95% confidence intervals. The dashed horizontal line (*y* = 0) indicates no change from the 1990 reference level.

Figure 4 presents trends in MHD AS-YLDs from 1990 to 2021 across different SDI levels and globally. A downward trend was observed in low and low–median SDI countries, with burden reductions of ~5 and 20%, respectively, by 2021. In contrast, median, high-median, and high SDI countries showed a rising burden after 2000, with a ~40%−50% increase by 2021 compared to 1990.

Globally, the trend was one of mild increase. Importantly, except for high–median SDI countries where the 95% confidence interval of the 2021 year effect did not cross zero, all other SDI groups showed non-significant year effects after adjusting for WBGT and other covariates. This indicates that the calendar year alone had limited explanatory power for changes in MHD AS-YLDs, emphasizing the stronger impact of heat exposure and environmental factors (Figure 4).

### 3.5 Heterogeneity of WBGT-MHD associations across climate zones

Restricted cubic spline analysis revealed substantial heterogeneity in the associations between WBGT and maternal hypertensive disorder (MHD) AS-YLDs across different SDI levels and climate zones (Supplementary Figure 3). In high-SDI temperate countries, the curve exhibited a *U*-shaped pattern, with the lowest burden at 9.2 °C and a peak around 12.2 °C. In contrast, in high-SDI subtropical countries, the risk rose sharply above 22.8 °C. In high- to middle-SDI settings, troughs appeared between 13.2 and 16.8 °C, followed by a gradual increase with rising temperature. In subtropical and tropical countries with middle and low-middle SDI, the curves showed a steep positive slope, with the highest burdens concentrated between 24.5 and 27.7 °C. Notably, middle-SDI subtropical countries displayed a *J*-shaped association, while most middle-SDI tropical countries clustered above 23 °C, where MHD AS-YLDs tended to decline with higher WBGT. In low-SDI subtropical countries, a similar *J*-shaped curve was observed, with burdens declining beyond 23.2 °C. In low-middle SDI tropical regions, a bimodal pattern emerged, with distinct peaks at 24.2 and 28.1 °C. Since WBGT generally exceeded 22 °C in low-middle and low-SDI tropical countries, the distributions predominantly followed *U*-shaped or bimodal trajectories, consistent with the global WBGT–MHD AS-YLDs relationship. Except for middle-SDI subtropical countries, where MHD AS-YLDs rose with WBGT and plateaued beyond 14.9 °C, the other RCS curves closely mirrored the global pattern, further supporting the robustness of our model.

## 4 Discussion

Wet-bulb temperature is a key parameter for assessing environmental heat stress. It integrates air temperature, humidity, and wind speed through the principle of evaporative cooling, providing a comprehensive index of the thermal load on the human body. Under high temperature and humidity, the body's capacity for heat dissipation is thereby severely challenged. Sweat evaporation becomes less effective, thus impairing thermoregulation and leading to rising core body temperature, which in turn triggers a cascade of physiological stress responses (25).

Pregnant women are particularly vulnerable in high heat and humidity environments due to the physiological adjustments required to support fetal growth. These include increased metabolic rate and endogenous heat production, rendering them less tolerant to high heat and humidity. Even small variations in wet-bulb temperature may disrupt their fragile thermal balance, thereby increasing the risk of maternal hypertensive disorders and other obstetric complications (26). Moreover, from a physiological perspective, elevated wet-bulb temperatures can alter endocrine function in pregnant women. Increased secretion of stress hormones, such as cortisol, may result in elevated blood glucose, water-sodium retention, and subsequent increases in blood pressure (27).

Using data spanning 1990 to 2021, this study systematically evaluated the relationship between wet-bulb globe temperature (WBGT) and years lived with disability (AS-YLDs) due to maternal hypertensive disorders (MHD) at the global scale. A significant nonlinear association was observed, with *U*-shaped or *J*-shaped exposure–response curves prominent in countries with low to high–median Sociodemographic Index (SDI). These findings suggest that heat exposure poses risks not only at extreme levels, but also through interactions with developmental, environmental, and healthcare-related factors. These interactions further vary by national context.

Firstly, global and SDI-stratified comparisons revealed that WBGT was highest in low SDI countries, where MHD AS-YLDs were also most severe. Conversely, high SDI countries had the lowest WBGT and burden. Despite higher environmental exposures (e.g., O_3_, NO_2_) and behavioral risks (e.g., tobacco and alcohol use) in high SDI settings, their MHD burden remained relatively low—indicating the potential protective roles of healthcare infrastructure, prenatal management, and social support systems in these regions (20, 28).

Secondly, restricted cubic spline (RCS) modeling confirmed the presence of *U*-shaped or *J*-shaped relationships between WBGT and MHD burden across most SDI groups. In low–median, median, high–median SDI countries, and at the global level, MHD AS-YLDs began to increase sharply after passing respective inflection points (e.g., 11.0, 13.4 and 11.1 °C), highlighting health risks from both heat and cold stress. In low SDI countries, where WBGT values were consistently above 14 °C, cold-related stress was negligible, while heat-related risks were prominent. Interestingly, in high SDI countries, the WBGT–MHD relationship appeared atypical and overall negatively associated—likely due to strong heat adaptation mechanisms such as widespread air conditioning, frequent antenatal care, and higher maternal education levels. Moreover, at very high WBGT levels (e.g., 22.8–27.2 °C), MHD AS-YLDs temporarily declined before rising again in low, low-median, median, high-median, and global trends—suggesting possible threshold and rebound effects.

The RCS models further validated this nonlinear association, showing that several spline terms of WBGT were significantly associated with MHD AS-YLDs. Additionally, SDI emerged as a robust protective factor. In contrast, other covariates such as BMI, tobacco, and alcohol use showed limited or inconsistent effects—supporting the notion that thermal exposure and development level are the primary global drivers of maternal hypertensive burden (29, 30).

Finally, while trends in year fixed effects from the RCS model (Figure 4) showed rising or falling MHD AS-YLDs in various countries, these trends largely lost statistical significance after controlling for WBGT and other exposures. This suggests that calendar time alone, independent of environmental changes, contributes little to the burden of maternal hypertension. Instead, the temporal dynamics and cumulative impacts of WBGT may carry greater biological relevance and policy implications.

To our knowledge, most existing studies on temperature and MHD focus on regional incidence rates and ambient temperature. Very few studies have investigated the association between global thermal indices and MHD YLDs. Our findings align with prior research. For instance, Xiong et al. reported that extreme temperatures increased the risk of hypertensive disorders of pregnancy (HDP) in multiple Chinese cities. Gebremedhin et al. analyzed over 415,000 pregnancies in Western Australia and found that both low (10.2 °C) and high (26.0 °C) UTCI (Universal Thermal Climate Index) exposures during early to mid-pregnancy were linked to higher odds of hypertensive disorders of pregnancy, with peak risks for gestational hypertension at 8–18 weeks and preeclampsia at 11–16 weeks (31). Similarly, Zeng et al. found that first-trimester exposure to extreme heat [aOR (adjusted Odds Ratio) = 1.24, 95% CI: 1.12–1.38] and moderate heat (aOR = 1.22, 95% CI: 1.10–1.35) was associated with increased preeclampsia risk (32). A South African study also revealed a significant association between mean weekly temperatures of 23 °C (vs. 18 °C) during gestational weeks 2–5 and elevated PEH risk (33). These studies provide strong empirical support for our conclusions.

Here we also discuss why the RCS curve shows a local decline beyond ~24 °C. A plausible explanation is behavioral and structural adaptation at very high heat: pregnant women are more likely to remain indoors, use air-conditioning or cooling centers, and reduce physical exertion; concurrently, heat-health warning systems and clinical triage can shift resources toward high-risk pregnancies. These “self-protection” and “system-level protection” responses can attenuate observed risk at the hottest WBGT levels. In addition, our outcome is MHD AS-YLDs (disease burden) rather than incidence. In settings with persistently high WBGT, differences in care access, diagnosis, and recording practices may shape burden estimates and contribute to non-monotonic patterns (as acknowledged in the Limitations). Finally, uncertainty bands widen in this range, so the post-24 °C decline should be interpreted as a local feature under increasing uncertainty rather than a strictly monotonic protective effect.

Future studies should set up large, forward-looking cohorts of pregnant women in tropical, temperate, and cold regions, covering both urban and rural areas. Throughout pregnancy, blood pressure and lab tests need to be recorded week by week, while key personal details—such as BMI, medical history, physical work load, air-conditioning use, diet, and physical activity—should be collected. Using multilevel or “target trial” designs, researchers can look at the short- and long-term effects of wet-bulb globe temperature (WBGT) during early, middle, and late pregnancy and across different types of hypertensive disorders (gestational hypertension, preeclampsia, eclampsia). These efforts will clarify the causal link between heat exposure and maternal hypertension at both individual and national levels, and provide solid evidence for targeted cooling measures and public-health policies in a warming world.

Taken together, our findings highlight the urgent need to address the impact of high-temperature environments on pregnancy, particularly in low- and middle-SDI countries facing escalating climate risks. Adaptive strategies should be tailored to local contexts. In low-SDI regions, priorities include improving maternal living environments, ensuring access to affordable cooling resources, and expanding basic maternal healthcare services. In middle-SDI settings, integrating heat-health early warning systems into prenatal care and increasing the frequency of antenatal visits during hot and humid seasons could help mitigate risks. In high-SDI countries, continuous surveillance and targeted support for vulnerable groups—such as women with obesity, advanced maternal age, or pre-existing hypertension—remain essential. Across all settings, early, personalized interventions including dietary counseling, moderate exercise, and mental health support can strengthen maternal resilience to heat exposure. These differentiated strategies will be crucial to safeguarding maternal and infant health in a warming world.

At the individual level, pregnant women should avoid prolonged exposure to extreme temperatures to reduce the risk of developing hypertensive complications.

## 5 Strengths and limitations

This study has several limitations that point the way for future research. First, because we relied on national-level annual summary data, we were unable to identify heat-sensitive windows during early, mid-, and late pregnancy or to examine different subtypes of maternal hypertensive disorders. Second, our models were constructed only at the SDI-group, climate-zone, and global levels, which may have overlooked country-specific differences in healthcare capacity and policy contexts. Third, the ecological, country—year design—constrained by the availability of annual GBD YLDs—precluded (i) inclusion of individual-level factors (e.g., parity, genetics, comorbidities, cooling behaviors) and (ii) analysis of short-term heat stress (e.g., heatwaves and intra-seasonal fluctuations), leaving room for residual confounding and potentially attenuating acute effects. Finally, even with year fixed effects, an observational ecological study cannot completely exclude the influence of unmeasured temporal trends or policy shifts, so causal interpretations should be made with caution.

At the same time, this study also has important strengths. Unlike most previous work that focused solely on ambient temperature, we employed the wet-bulb globe temperature (WBGT)—a composite indicator that reflects temperature, humidity, solar radiation, and wind speed—providing a more physiologically relevant and comprehensive assessment of heat exposure. We integrated multiple high-quality global datasets (GBD, CHC, and ERA5) and applied bias adjustment to achieve robust and comparable exposure estimates across countries, allowing us to explore the relationship between WBGT and maternal hypertensive disorders from a global perspective. By using years lived with disability (YLDs) as the outcome, we were able to capture not only mortality but also disability burden, thereby offering a more complete picture of the threat that heat exposure poses to women's health. Finally, stratified analyses by SDI levels and climate zones identified vulnerable populations and provided actionable evidence for targeted public health interventions.

## 6 Conclusion

This study demonstrates a significant nonlinear association between WBGT and the disability burden of maternal hypertensive disorders, with *U*- or *J*-shaped patterns particularly evident in low- to median-SDI countries. The heterogeneity observed across SDI levels and climate zones underscores the importance of adaptation capacity in shaping vulnerability. To mitigate these risks, context-specific strategies are required: expanding maternal healthcare access and affordable cooling measures in low-SDI regions, integrating heat-health early warning systems into maternal health programs in middle-SDI countries, and maintaining surveillance with targeted support for disadvantaged groups even in high-SDI settings. These tailored actions will be critical to protecting maternal and infant health in a warming world.

## Data Availability

The original contributions presented in the study are included in the article/Supplementary material, further inquiries can be directed to the corresponding author.

## Supplementary material

Supplementary Figure 1 Annual mean wet-bulb globe temperature (WBGT) for China and India, 1990–2021. The line chart displays the annual mean WBGT for China (red) and India (cyan) from 1990 to 2021. The *y*-axis represents WBGT (°C), and the *x*-axis shows calendar years. The black dashed vertical line at 2016 marks the transition point between data sources: CHC data were used for years prior to 2017, and ERA5 data—adjusted for country-level bias—were used from 2017 onward. The smooth continuity of the curves across the breakpoint indicates no structural discontinuity after data merging.

Supplementary Figure 2 Forest plot of global associations with age-standardized YLDs for maternal hypertensive disorders (MHD AS-YLDs). The *x*-axis shows the estimated percent change in MHD AS-YLDs per unit increase in each covariate, along with corresponding 95% confidence intervals. The *y*-axis lists the WBGT spline terms (knot coefficients) and additional covariates (reference year: 1990). Red circles indicate point estimates; blue horizontal lines represent 95% confidence intervals. The vertical dashed line indicates the null effect (0% change). Confidence intervals entirely to the right (left) of the null line suggest a statistically significant positive (negative) association with MHD AS-YLDs.

Supplementary Figure 3 Restricted cubic spline (RCS) curves for the association between wet-bulb globe temperature (WBGT, °C) and age-standardized YLDs for maternal hypertensive disorders (MHD AS-YLDs, per 100,000) across SDI–climate zone strata, 1990–2021. The figure presents stratified RCS curves by sociodemographic index (SDI) group and climate zone. Each panel shows the adjusted relationship between annual mean WBGT (°C, *x*-axis) and MHD AS-YLDs (*y*-axis). Red solid lines represent multivariable-adjusted RCS fitted values, with golden shaded areas indicating 95% confidence intervals. Blue and red dots mark inflection points where the slope changes sign, corresponding to local minima or maxima of MHD burden. Text labels indicate the WBGT inflection temperatures and the estimated MHD AS-YLDs at those points. These inflection points highlight critical thresholds where maternal health burden shifts direction, reflecting heterogeneous WBGT–MHD associations across SDI and climate zone strata.

Supplementary Table 1 Comparison of model fit between linear and restricted cubic spline (RCS) terms for WBGT in Predicting Age-Standardized YLDs for Maternal Hypertensive Disorders (MHD AS-YLDs) Across SDI Groups and Globally. This table presents the goodness-of-fit comparison between models using linear terms vs. RCS terms for WBGT across different Sociodemographic Index (SDI) groups and at the global level. Metrics include residual sum of squares (RSS) and *F*-statistics for evaluating model improvement.

Supplementary Table 2 Comparison of model fit (AIC/BIC) for the association between WBGT and MHD AS-YLDs. Linear, quadratic, 3rd-degree polynomial, RCS (3- and 4-knot), and GAM models were fitted with the same covariates. Lower values indicate better fit.

Supplementary Table 3 Variance inflation factors (VIF) for covariates. Variance inflation factors were calculated for all covariates included in the fully adjusted models to assess potential multicollinearity. All VIF values were below the commonly accepted threshold of five, suggesting that the covariates were sufficiently independent and that multicollinearity was unlikely to bias coefficient estimates. This finding confirms the robustness of the regression results and supports the validity of including socioeconomic, behavioral, and environmental factors simultaneously in the models.
