# Supplementary figures and images for: Nonlinear association between wet-bulb globe temperature and maternal hypertensive disorders burden: a global analysis from 1990 to 2021
Source: Front Public Health. 2025 Oct 10;13:1678469. doi: 10.3389/fpubh.2025.1678469 (PMC12549697; doi:10.3389/fpubh.2025.1678469)

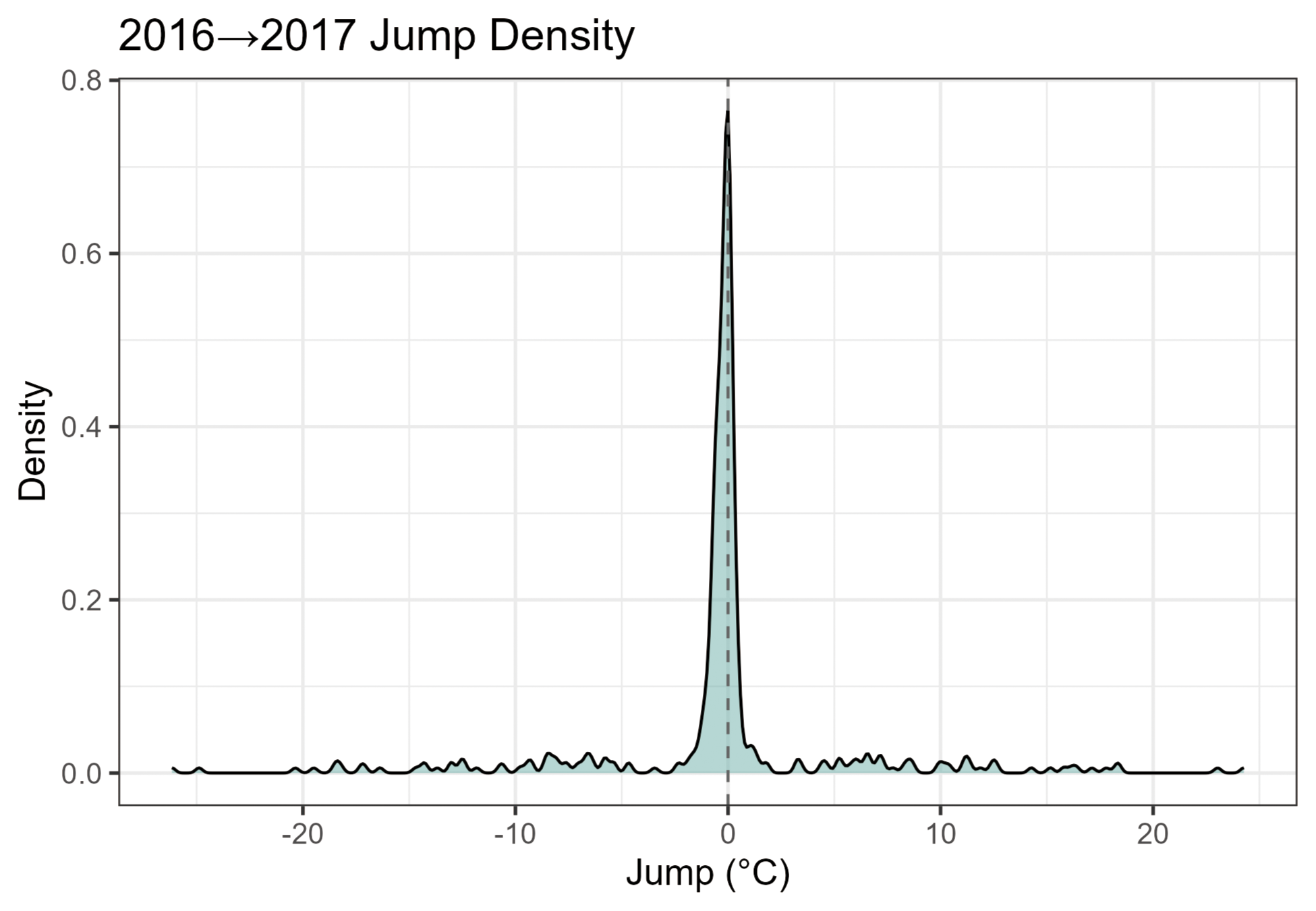

Supplement: Supplementary Figure 1 — Annual mean wet-bulb globe temperature (WBGT) for China and India, 1990–2021. The line chart displays the annual mean WBGT for China (red) and India (cyan) from 1990 to 2021. The y-axis represents WBGT (°C), and the x-axis shows calendar years. The black dashed vertical line at 2016 marks the transition point between data sources: CHC data were used for years prior to 2017, and ERA5 data—adjusted for country-level bias—were used from 2017 onward. The smooth continuity of the curves across the breakpoint indicates no structural discontinuity after data merging. [file Image_1.TIF]

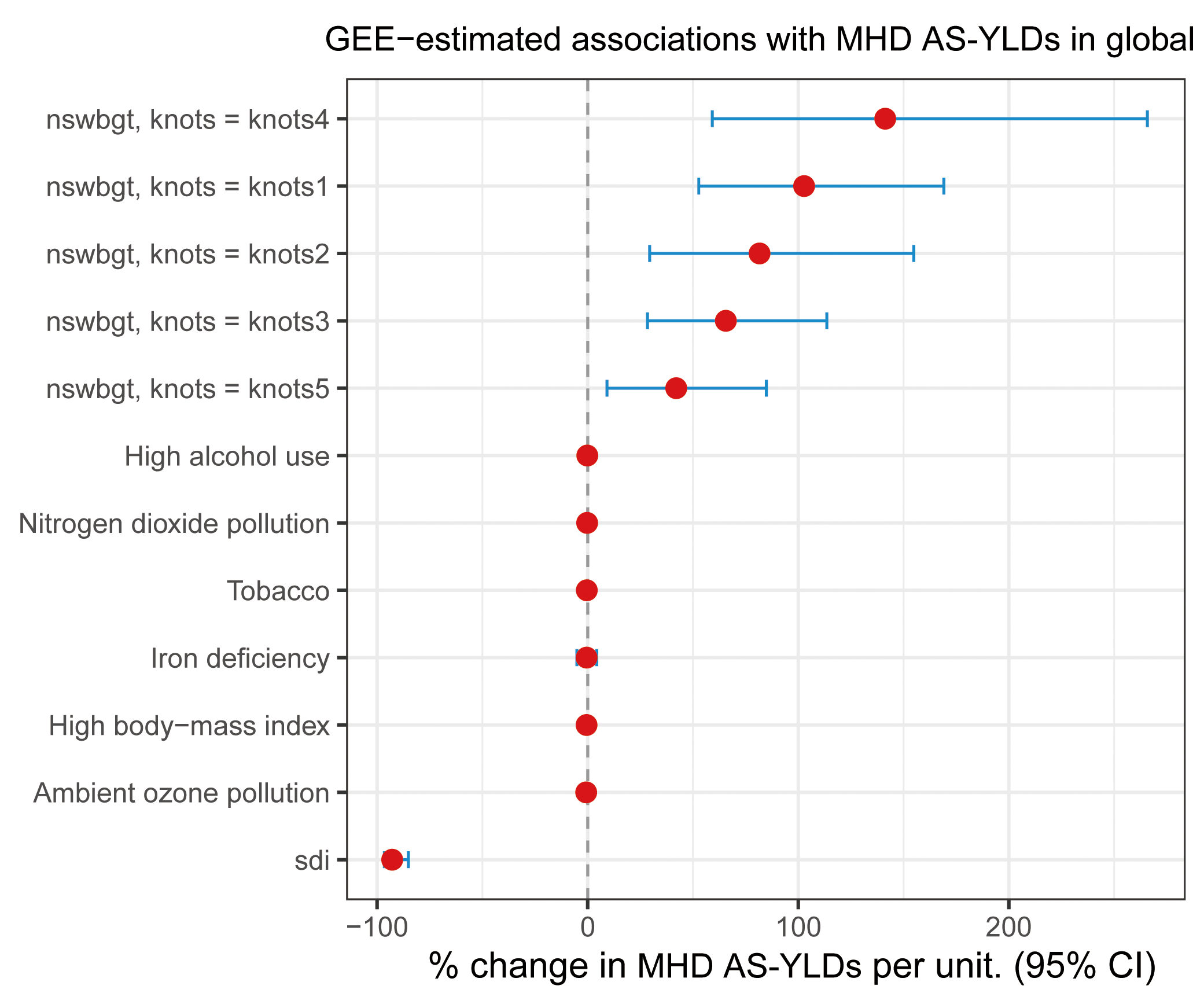

Supplement: Supplementary Figure 2 — Forest plot of global associations with age-standardized YLDs for maternal hypertensive disorders (MHD AS-YLDs). The x-axis shows the estimated percent change in MHD AS-YLDs per unit increase in each covariate, along with corresponding 95% confidence intervals. The y-axis lists the WBGT spline terms (knot coefficients) and additional covariates (reference year: 1990). Red circles indicate point estimates; blue horizontal lines represent 95% confidence intervals. The vertical dashed line indicates the null effect (0% change). Confidence intervals entirely to the right (left) of the null line suggest a statistically significant positive (negative) association with MHD AS-YLDs. [file Image_2.TIF]

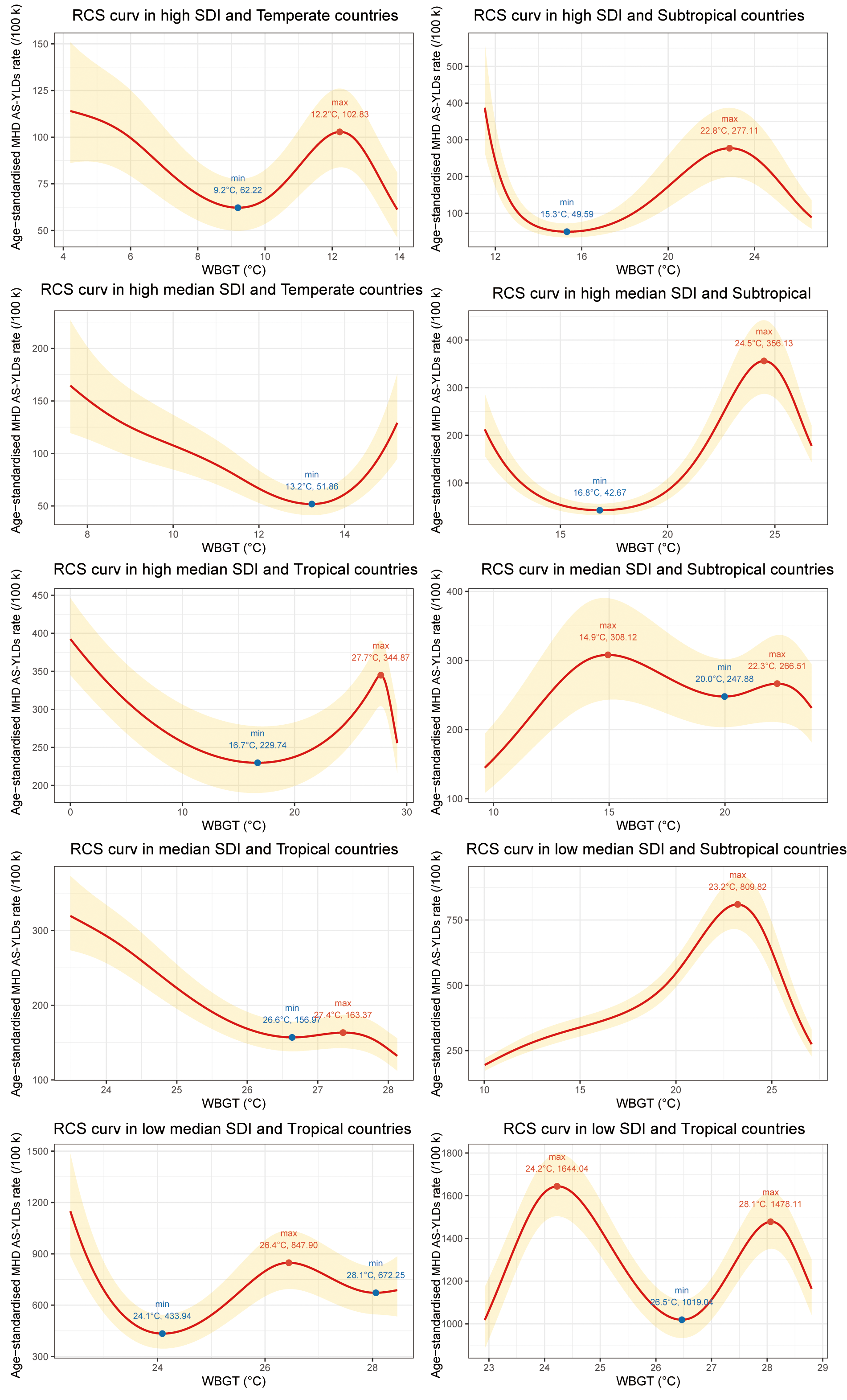

Supplement: Supplementary Figure 3 — Restricted cubic spline (RCS) curves for the association between wet-bulb globe temperature (WBGT, °C) and age-standardized YLDs for maternal hypertensive disorders (MHD AS-YLDs, per 100,000) across SDI–climate zone strata, 1990–2021. The figure presents stratified RCS curves by sociodemographic index (SDI) group and climate zone. Each panel shows the adjusted relationship between annual mean WBGT (°C, x-axis) and MHD AS-YLDs (y-axis). Red solid lines represent multivariable-adjusted RCS fitted values, with golden shaded areas indicating 95% confidence intervals. Blue and red dots mark inflection points where the slope changes sign, corresponding to local minima or maxima of MHD burden. Text labels indicate the WBGT inflection temperatures and the estimated MHD AS-YLDs at those points. These inflection points highlight critical thresholds where maternal health burden shifts direction, reflecting heterogeneous WBGT–MHD associations across SDI and climate zone strata. [file Image_3.TIF]
